# Implementing the Federal Smoke-Free Public Housing Policy in New York City: Understanding Challenges and Opportunities for Improving Policy Impact

**DOI:** 10.3390/ijerph182312565

**Published:** 2021-11-29

**Authors:** Nan Jiang, Emily Gill, Lorna E. Thorpe, Erin S. Rogers, Cora de Leon, Elle Anastasiou, Sue A. Kaplan, Donna Shelley

**Affiliations:** 1 Department of Population Health, Grossman School of Medicine, New York University, New York, NY 10016, USA; emily.gill@nyulangone.org (E.G.); lorna.thorpe@nyulangone.org (L.E.T.); erin.rogers@nyulangone.org (E.S.R.); elle.anastasiou@nyulangone.org (E.A.); sue.kaplan@nyulangone.org (S.A.K.); 2 Silver School of Social Work, New York University, New York, NY 10003, USA; cora.deleon@nyu.edu; 3 School of Global Public Health, New York University, New York, NY 10003, USA; donna.shelley@nyu.edu

**Keywords:** smoke-free policy, public housing, implementation

## Abstract

In 2018, the U.S. Department of Housing and Urban Development required public housing authorities to implement a smoke-free housing (SFH) policy that included individual apartments. We analyzed the policy implementation process in the New York City Public Housing Authority (NYCHA). From June–November 2019, we conducted 9 focus groups with 64 NYCHA residents (smokers and nonsmokers), 8 key informant interviews with NYCHA staff and resident association leaders, and repeated surveys with a cohort of 130 nonsmoking households pre- and 12-month post policy. One year post policy implementation, participants reported widespread smoking violations and multi-level factors impeding policy implementation. These included the shared belief among residents and staff that the policy overreached by “telling people what to do in their own apartments”. This hindered compliance and enforcement efforts. Inconsistent enforcement of illegal marijuana use, staff smoking violations, and a lack of accountability for other pressing housing issues created the perception that smokers were being unfairly targeted, as did the lack of smoking cessation resources. Resident support for the policy remained unchanged but satisfaction with enforcement declined (60.1% vs. 48.8%, *p* = 0.047). We identified multilevel contextual factors that are influencing SFH policy implementation. Findings can inform the design of strategies to optimize policy implementation.

## 1. Introduction

Exposure to secondhand smoke (SHS) is associated with significant adverse health effects and more than 40,000 deaths annually in the U.S. [1,2] Despite large declines in SHS exposure over the last two decades, 58 million nonsmokers are still routinely exposed, primarily at home [1,3]. Home exposure is highest among low-income and racial/ethnic minorities, reflecting higher smoking prevalence and differential risks across housing environments. Residents in public housing report a disproportionately high smoking rate than the general U.S. adult population (33.6% vs. 16.8%) [4] and they are more likely to live in multiunit housing, which place this group at elevated risk of exposure to SHS [5,6,7,8].

To reduce health disparities related to SHS exposure, the U.S. Department of Housing and Urban Development (HUD) issued a rule requiring public housing authorities (PHAs) to implement a smoke-free housing (SFH) policy in July 2018 [9]. The policy prohibits smoking in all indoor spaces, including individual residences and outdoor areas within 25 feet of buildings. Existing literature has concluded that the majority of multiunit housing residents support smoke-free building policies [10]. With more than 2 million people living in public housing, the potential public health impact of the SFH policy is substantial [11].

We are conducting a longitudinal natural experiment to assess the impact of HUD’s SFH policy on SHS exposure among residents living in New York City Housing Authority (NYCHA), the nation’s largest PHA. Using state-of-the art air monitoring procedures, 12 months after the policy came into effect, we found little change in the airborne nicotine concentration and particulate matter (PM_2.5_) in nonsmoker apartments and common areas of NYCHA buildings compared with baseline measures [12]. The small number of studies that have examined air quality impacts of SFH policy have similarly demonstrated a modest and/or non-significant reduction in SHS exposure [13,14,15,16,17].

These data emphasize the complexity of implementing SFH policies, yet little empirical evidence exists to guide this process, including the selection of strategies to improve implementation effectiveness [18,19]. This paper presents findings from our study’s concurrent analysis of the policy implementation process and is the first study to elucidate the multi-level barriers to and facilitators of HUD’s SFH policy compliance and enforcement.

## 2. Materials and Methods

### 2.1. Conceptual Framework

**Figure 1:**
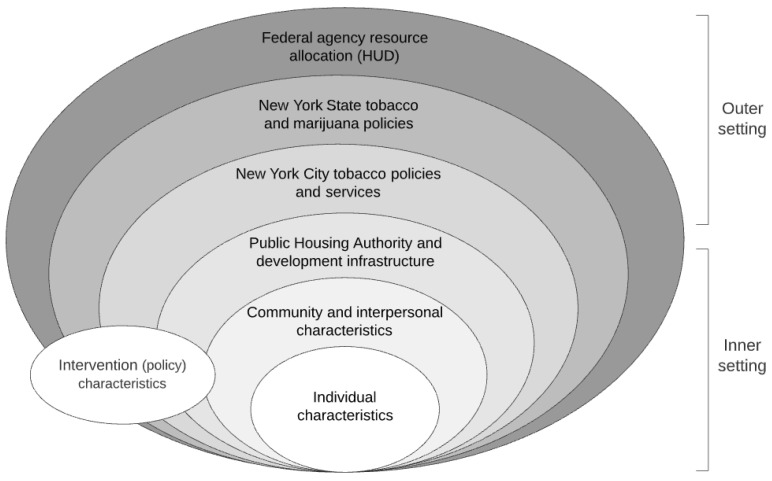
CFIR and SEM integrated framework.

The study relied on two frameworks, the consolidated framework for implementation research (CFIR) and the socioecological model (SEM). The CFIR characterizes contextual determinants of implementation across five domains. These include: (1) the outer setting (e.g., HUD resource allocation and external policies such as New York State marijuana policy); (2) the inner setting (e.g., community and interpersonal characteristics such as interactions between smokers and nonsmokers and between residents and PHA staff, and infrastructure such as building quality); (3) individual characteristics (e.g., knowledge and attitude); (4) intervention characteristics (e.g., complexity); and (5) implementation process (e.g., engagement and enforcement) [20]. The SEM also considers the influence of multi-level factors on effective policy implementation [21] but expands the contextualization of individual characteristics and behaviors to include interpersonal interactions, community interactions, and community infrastructure. Figure 1 places the CFIR within the ecological framework to make explicit the interaction across domains and that federal, state, and city policies may interact in ways that may support or impede policy implementation.

### 2.2. Qualitative Data Collection

The study was approved by New York University Grossman School of Medicine’s Institutional Review Board. We collected qualitative data from June through to November 2019 (12–18 months post policy implementation). A total of 9 focus groups (FGs) were conducted with 64 residents that were recruited from 10 NYCHA buildings in 4 developments that were engaged in the larger longitudinal data collection. Details of the longitudinal study design are published elsewhere [22]. Approximately 15.7% of our longitudinal study participants from the 10 NYCHA buildings reported current (past 30-day) smoking before the SFH policy went into effect [22]. We aimed to recruit both smokers and nonsmokers who spoke English or Spanish to the FGs. A total of 4 FGs were conducted in English with nonsmokers (*n* = 30), 4 in English with smokers (*n* = 26), and 1 in Spanish with nonsmokers (*n* = 8). Eligibility criteria included: (1) has lived in one of the four developments for ≥1 year, (2) ≥18 years old, and (3) English or Spanish speaker. Participants were recruited via flyers distributed in developments and in collaboration with community-based organizations working with NYCHA residents. Additionally, we conducted semi-structured phone interviews with eight key informants (KIs): three resident association (RA) leaders and five NYCHA staff (leadership and building managers who were responsible for enforcing the policy).

FGs and KI interviews were guided by the CFIR and SEM and explored participants’ perceived barriers and facilitators to policy implementation for each of the five domains described in the conceptual framework section. Specifically, we started with questions about community and interpersonal characteristics (e.g., “what do you like and dislike about this development”?) and individual characteristics (e.g., attitudes and knowledge about the policy). Next, we asked participants’ perceptions about policy enforcement and compliance, factors that influenced the implementation process (e.g., marijuana policy, intervention complexity, structural barriers, community and interpersonal norms, smoking cessation resources, resident engagement and enforcement). FGs and KI interviews also elicited suggestions for strategies to address current challenges to implementing the SFH policy. FGs and KIs were audio-taped and transcribed verbatim. We used the Standards for Reporting Qualitative Research (SRQR) as a checklist to report qualitative findings (Table S1).

### 2.3. Resident Surveys

We enrolled 157 nonsmoking households in our larger longitudinal study in order to assess SFH policy’s impact on secondhand smoke exposure among NYCHA residents. Nonsmoking households were defined as households where no members currently smoked cigarettes or used other tobacco products. One adult member of the household was selected to complete the surveys. Detailed information on the study population and procedures has been described elsewhere [22]. Among the 157 nonsmoking households, 153 completed a baseline survey before the SFH policy went into effect (April–July 2018), and 130 completed a 12-month follow-up survey (May–September 2019).

The survey assessed residents′ support for the policy (“how would you describe your current level of support for NYCHA smoke-free homes policy”?) and satisfaction with policy implementation (“how satisfied are you with how NYCHA has introduced the new policy about smoking” and “how satisfied are you with how your development has enforced the current policy about smoking”?). Responses were dichotomized into “support”/“don’t support” or “satisfied”/“not satisfied”. The survey also assessed exposure to SFH policy-related programming and communication strategies and whether residents submitted a complaint about policy violations in the past 6 months.

### 2.4. Data Analysis

Qualitative data analyses were conducted using NVivo 12. Using both a deductive approach (guided by CFIR and SEM domains in the qualitative guide) and an inductive analytic approach [23,24], four team members independently read a subset of transcripts to identify preliminary themes, relevant patterns, and generative questions. Coders met to review findings, discussed clustered concepts, and identified emergent themes and subthemes. This iterative process continued until the team reached consensus and generated a final codebook. Quantitative survey data were analyzed using SAS 9.4. Descriptive statistics summarized participants’ perceptions. A McNemar test was performed to compare the change for paired binomial data.

## 3. Results

### 3.1. Qualitative Findings

Table 1 shows FG participant characteristics. Overall, compliance with and enforcement of the SFH policy was described as poor. Both smokers and nonsmokers reported no post-policy changes in smoking behaviors, noting that “*people are still smoking in their apartments, in the hallways, and in the stairwell*”, and “*it*’*s as if the policy doesn’t even exist*”. The findings are organized by CFIR domains. Subthemes represent the most frequently cited multilevel barriers to policy compliance and enforcement. Table 2 provides representative quotes by domain.

#### 3.1.1. Outer Setting

Barriers in this domain included a lack of external resources at the federal and local levels to support implementation, and state marijuana policies that were conflicting with the SFH policy. Additional efforts to engage external partners (e.g., NYC Department of Health and Mental Hygiene) was described as facilitating implementation.

##### HUD Resources

NYCHA leaders described receiving guidance materials from HUD, but HUD did not provide funding for implementation strategies (e.g., resident engagement, communication).

##### PHA Partnerships

Without HUD’s financial support, PHA staff described leveraging community partnerships to obtain resources for a new smoking cessation program (e.g., American Lung Association (ALA)) and to strengthen the integration of the SFH policy into NYCHA’s other health and housing initiatives.

##### Marijuana Policy

Residents described marijuana use in and outside of buildings as pervasive. Some described exposure to marijuana smoke as more common than SHS exposure. At the time this study began, marijuana was illegal in New York State. Widespread use was attributed to loosening enforcement of the law. Smokers and nonsmokers agreed that ignoring marijuana use while enforcing the SFH policy created a double standard that further undermined smoker compliance.

#### 3.1.2. Intervention Characteristics

The HUD’s SFH policy extended the no-smoking ban to include private apartments, which created barriers to policy enforcement and compliance. Residents and staff uniformly viewed the policy as an infringement on the right to privacy. Smokers believed that the policy violated their right to act autonomously in their own homes and were thus disinclined to comply. Nonsmokers largely supported the policy, but they shared smokers’ concerns about a policy that dictates what residents can and cannot do in their own homes. Similarly, staff, whose responsibility was to enforce the policy, believed that it was an invasion of privacy to tell a resident that they could not smoke at home.

#### 3.1.3. Individual Characteristics

Nonsmokers’ support for the policy was largely based on the potential health benefits. A few smokers also acknowledged that the “*policy can be a well-meaning policy, just for nonsmoking residents*”. Although residents were aware of the policy, most were unfamiliar with the enforcement process. For example, residents perceived the risk of eviction was high. A smoker suggested that if “*you don’t stop smoking, you get evicted*”. This belief reduced support for the policy among smokers and nonsmokers. However, NYCHA has a multi-step approach to address policy violations that is designed to avoid evictions. This includes multiple warnings and smoking cessation support.

#### 3.1.4. Inner Setting

The inner setting is defined broadly to reflect both the characteristics of public housing (e.g., building quality), the community and interpersonal interactions between smokers and nonsmokers and between residents and PHA staff.

##### Structural and Environmental Barriers

Smokers provided examples of challenges that arose from broken elevators and adverse weather conditions that precluded them from going outside to smoke. The density of NYCHA developments also made it difficult to meet the requirement to refrain from smoking within 25′ of a building.

##### Interpersonal Safety Concerns

Nonsmokers and building staff described feeling unsafe about approaching smokers who were violating the policy. Nonsmokers were concerned that reporting a violation would create distrust and hostility among neighbors or lead to retaliation. Others explained that they “*wouldn’t dare ask somebody to not smoke*”. Staff responsible for enforcing the policy also reported that they were advised against ever “*confronting anybody*”.

##### Community Cohesiveness

There was variation in how residents described the community characteristics of their developments, which have several buildings. Some described their developments as supportive, “*family oriented*”, and cohesive, particularly in buildings where “*everybody’s been here for ages*” and “*knows each other*”. In contrast, other residents described changes in their community over time that were negatively affecting resident interactions and created an environment in which it was “*every man for themselves atmosphere with many who don’t even bother with rules*”. For example, a general lack of consideration for neighbors’ health in these settings was manifested by smokers continuing to smoke in close proximity to nonsmokers even after they were asked to move. These residents similarly described growing safety concerns, and a lack of trust among residents that negatively impacted nonsmokers’ willingness to report violations.

##### Lack of Smoking Cessation Support

A lack of cessation support was viewed as a major barrier to compliance. Nonsmokers and staff sympathized with the need for easy to access cessation resources, including support groups or “*centers where those who smoke can receive more services*”.

##### Relative Priority of SFH Policy

Smokers and nonsmokers believed that smoking was receiving a disproportionate amount of attention compared with other housing concerns that were not being addressed. Nonsmokers noted that “*there’re a lot of things inside the apartment that are not being dealt with*”. Smokers noted the discrepancy between the potential serious consequences (i.e., eviction) of smoking violations compared with the lack of accountability for other housing policy violations. From their perspective, this provided an additional rationale for ignoring the SFH policy.

The PHA was responsible for ongoing implementation of a range of healthy and safe homes initiatives and programs, including addressing mold, lead safety and linking residents to health care services. In the context of this large health promotion agenda, the relative lack of resources specifically allocated for SFH created implementation and sustainability challenges.

##### Compatibility

Staff were not always clear about their role in enforcing the policy and reported a large number of competing priorities that at times felt “*overwhelming*” and may have prevented them from focusing on the enforcement.

#### 3.1.5. Implementation Process

##### Planning and Community Engagement

NYCHA created an advisory board to develop their final policy and implementation strategies. These efforts informed the launch of the smoke-free NYCHA initiative and defined an implementation plan that included a range of operational and programmatic efforts (e.g., communication strategies) needed to support the roll-out. NYCHA also initiated a year-long process of resident engagement prior to the launch date. This included numerous meetings held across developments to explain the policy and enforcement processes. However, most residents were not aware of these efforts and felt that the process “*could have worked better*” with greater engagement and more information sharing. Smokers, in particular, described feeling out of “*the loop*”.

##### Enforcement

Nonsmokers reported that the policy was not being adequately enforced. Staff suggested several reasons for gaps in enforcement that included a lack of clarity about their roles and responsibilities in relation to the policy, a lack of time to address violations, and as noted above, safety concerns about confronting smokers. Staff also raised questions about the feasibility of enforcing the policy when they were not willing to go into someone’s home “*to see whether or not they*’*re smoking*”. Nonsmokers were similarly reluctant to intervene because of safety concerns and, again, the shared belief in smokers’ right to smoke in their home.

Most residents described seeing staff smoking in nonsmoking areas and attributed this to their lack of motivation to intervene when they observed smokers violating the policy. From the smokers’ perspective, staff policy violations diminished their credibility and authority to “*tell them not to smoke*” and further reduced smokers’ motivation to comply.

#### 3.1.6. Strategies for Improving SFH Policy Implementation

Staff described strategies that they were implementing to address many of the challenges that residents described. For example, to address knowledge gaps and change attitudes, they described “*creating more communication content on how residents could get support for quitting and focusing on why this policy is so important. And how it affects their neighbors*”. They also described plans to integrate the SFH policy into “*other healthy-home programs like pest management*”, and to “*activate partnerships that help create healthier indoor environments*”. Other changes staff described included expanding the SFH policy working group to integrate other departments that are responsible for health and housing initiatives: “*With these new departments there*’*s a person who*’*s responsible for indoor air quality within the environmental health and safety team. We are going to be working with her to make sure that smoke-free is also factored into that*”. Staff also described the potential use of data to direct resources to the highest risk communities: “*We’ve got some data on which developments have the highest rates of avoidable hospitalizations for asthma*”. The staff described continuing to engage with resident leadership to gather information about how they can improve enforcement and compliance. Residents advocated for greater community engagement in the implementation process, with specific efforts to include smokers, and greater dissemination of information about enforcement procedures.

### 3.2. Survey Findings

Survey participants included 73% female, 53% Hispanic or Latino and 47% Non-Hispanic black (data not shown in the table). Participants were 51 years old (SD = 16.6). Support for the policy remained unchanged before and 12-month after the policy was launched (94.8% vs. 96.2%, *p* = 0.655), but satisfaction with policy enforcement decreased (60.1% vs. 48.8%, *p* = 0.047; Table 3).

## 4. Discussion

The present study examined the process of SFH policy implementation in the largest housing authority in the U.S. Findings may inform strategies for optimizing implementation process not only in the U.S., but also in other countries where enforcement of smoke-free policies is weak and research on effective approaches to improve compliance is lacking [25]. One year after the SFH policy went into effect, NYCHA residents and staff reported widespread smoking violations. The findings are consistent with our one-year pre-post longitudinal analysis of trends in objectively measured indoor exposure to SHS levels in NYCHA buildings, which demonstrated little change in air nicotine and PM_2.5_ in nonsmokers’ households and common areas [12].

We identified a range of factors that impeded policy compliance and enforcement. These included inner setting characteristics such as safety concerns that contributed to reluctance among staff and nonsmokers to approach smokers, and residents’ frustration with the lack of attention to other housing concerns. Hernández et al. [18] invoked the “social contract” framework in explaining why resident dissatisfaction with housing conditions would threaten SFH policy compliance. From the residents’ perspective, the lack of responsiveness to issues such as housing quality, community safety, and widespread marijuana use was viewed as a violation of this implicit contract, and thus, undermined the PHA’s authority to impose a SFH policy. Consequently, this reduced smokers’ motivation to comply and nonsmokers’ inclination to intervene or advocate for more consistent enforcement efforts.

The characteristics of the SFH policy, which was extended to include individual residences, also created challenges by changing the typical dynamic between smokers and nonsmokers, the latter of whom largely support the policy. Rokicki et al. [26] found that when public housing residents were asked to choose between residents’ right to smoke at home and residents’ right to breathe air free of SHS at home, residents chose the latter as more important. Similarly, nonsmokers overwhelmingly supported the SFH policy. However, residents and staff were aligned with smokers in believing that the revised SFH policy overreached by infringing on personal freedom. In studies conducted prior to the HUD SFH policy, residents living in public and affordable housing in Oregon, Massachusetts, and New York raised similar objections to extending the SFH policy to include private residences [18,19,27,28,29].

These data demonstrate a need for a better understanding of the trade-offs the new policy presented and of residents’ beliefs about the right to smoke which may vary depending on how the SFH policy is defined. In addition, an effective implementation process will require ongoing engagement with smokers to generate strategies that balance the policy requirements with smokers’ needs. Specifically, residents emphasized the need for easy access to smoking cessation services to improve successful policy implementation. This is consistent with evaluations of other SFH policies [19].

PHAs have the opportunity to leverage population-based smoking cessation programs to expand treatment access, such as the federal text messaging program, statewide Quitlines, and synergistic policies such as Medicaid coverage for cessation pharmacotherapy [30]. Strengthening partnerships with local health departments and other organizations such as the ALA may offer additional resources to facilitate access to a range of treatment options. Without these services, the community will continue to view the SFH policy as unfair to smokers. PHAs also have the autonomy to adapt the policy to local context.

Our study is the first to highlight the role of local marijuana laws in undermining the SFH policy which was developed at the federal level. NYCHA residents reported that exposure to marijuana smoke was often greater than cigarette smoke [31]. With the loosening of enforcement in states where marijuana use remains illegal, and the spread of legalization across the U.S., it is likely that other PHAs are experiencing a rise in the prevalence of indoor exposure to marijuana smoke (which is currently not included or enforceable under the HUD’s SFH policy). Since conducting this analysis, New York State has legalized marijuana use. PHAs in states such as New York may now have the authority to adapt their SFH policy to include marijuana. HUD should consider modifying their guidance documents to offer support to PHAs that exist within a range of marijuana policy contexts.

Changing marijuana laws demonstrate the challenge associated with implementing policies, such as SFH, in the context of ever changing social and legal systems. Unanticipated conflicts between policies, and unintended consequences that follow, are not systematically reported, and thus little is known about how to evaluate and respond to them. While current implementation science frameworks [32] commonly recommend analysis of external factors (e.g., policies, incentives) that influence the implementation of an evidence-based intervention, they are not explicit in modeling potential interactions *across policies* that may act at federal, state and local levels. Research is needed to develop theories of change for policy implementation that suggest both determinants of effective implementation and that guide the analysis of the impact of other policies, both positive and negative, on the implementation process and outcomes.

The implications of the marijuana policy for SFH policy implementation, and the other barriers to implementation that emerged in this analysis, further underscore the importance of creating an infrastructure for PHAs to monitor the implementation process to identify and respond to the wide range of contextual factors, including other conflicting and complementary policies (e.g., Medicaid coverage for smoking cessation medications) that are influencing SFH policy compliance and enforcement [33]. HUD did not provide resources to create or enhance PHA systems to monitor the policy. Partnerships between PHAs and academic institutions provide a model for leveraging multidisciplinary research and stakeholder expertise to support this process.

The current study demonstrated the value of a collaborative research effort, which included ongoing feedback from the PHA’s own assessments and engagement activities across a broad range of stakeholder groups, and provided opportunities for an iterative process of data collection, reflection, and adaptation as new information is obtained. For example, NYCHA has adapted their implementation strategies to include additional employee training, activities to further engage resident leaders, streamlining the enforcement process, efforts to ensure that signage is in place in all resident developments, and the launch of the Smoke-Free NYCHA liaison program. Liaisons provide information about free cessation resources and treatment and build partnerships with local stakeholders to support Smoke-Free NYCHA goals. They recently launched the ALA smoking cessation program. These, and the strategies suggested by residents, align with Leeman et al.’s classification of implementation strategies [34]. These include dissemination (e.g., communication with residents through multiple channels), capacity building (e.g., staff training), process (e.g., resident engagement and partners), and integration (e.g., liaison smoking cessation service). Technical assistance (capacity building) and additional resource allocation from local and federal agencies are needed to support these strategies. Scale up across the large number of very diverse developments is a challenge, but NYCHA’s cross department working group is supporting integration of the policy implementation process across the agency.

Improving implementation will also require strategies that address social and environmental barriers to implementing the SFH policy. Smokers and nonsmokers’ comments suggested opportunities to increase resident buy-in and foster a shared commitment to improving the health of all residents by creating space for both groups to interact and engage with leadership about other health and housing priorities. Extending cessation support to staff and creating mechanisms for enforcing compliance among staff who smoke are equally important policy implementation strategies.

This study has several limitations. First, participants were recruited from NYCHA developments in Upper Manhattan and may not generalize to other areas within NYC. Moreover, the disproportionally large number of public housing units in NYC creates unique challenges for policy implementation. Thus, findings may not be generalizable to other PHAs or elsewhere. However, our findings are generally consistent with emerging studies in other public or affordable housing properties [18,19,26,28,35]. Second, our surveys were conducted among nonsmokers only, and we lacked similar data on satisfaction with the SFH policy from smokers. Last, we examined the policy implementation process only one year after SFH policy went into effect. Plans for longitudinal data collection will provide additional information about what types of implementation strategies are feasible, acceptable, affordable, and can increase the effectiveness of policy implementation.

Our theory-driven assessment evaluated both the process and determinants of implementation, and their interaction across domains. Future research, conducted with early input from those affected by policies, is needed to expand our understanding of outer setting (e.g., intersecting state policies, partnerships), inner setting (e.g., community infrastructure and capacity), and individual determinants of policy implementation, what strategies are effective in addressing specific barriers at multiple levels, and how implementation strategies function in a range of PHA contexts.

## 5. Conclusions

Residents and staff described a complex combination of interacting, multi-level factors that are influencing policy implementation. These findings are informing adaptation and design of strategies aimed at optimizing SFH policy implementation. Research is needed to further explore this process and identify evidence-based strategies for optimizing policy implementation and public health outcomes.

## Figures and Tables

**Table 1:** Sample characteristics of focus group participants (N = 64).

	Development 1		Development 2		Development 3		Development 4		Total	
	*n*	(%)	*n*	(%)	*n*	(%)	*n*	(%)	*n*	(%)
Gender										
Male	8	(40.0)	2	(12.5)	2	(22.2)	2	(11.1)	14	(21.9)
Female	12	(60.0)	14	(87.5)	7	(77.8)	16	(88.9)	49	(76.6)
Mean age, Year (SD)	58.1	(13.8)	63.7	(10.1)	52.4	(20.8)	64.2	(15.2)	60.3	(15.1)
Smoking status										
Current smoker	10	(50.0)	7	(43.8)	5	(55.6)	4	(21.1)	26	(40.6)
Non-current smoker	10	(50.0)	9	(56.3)	4	(44.4)	15	(78.9)	38	(59.4)
Race/ethnicity										
Non-Hispanic white	3	(18.8)	1	(6.3)	2	(22.2)	11	(61.1)	17	(26.6)
Non-Hispanic black	6	(37.5)	15	(93.8)	7	(77.8)	5	(27.8)	33	(51.6)
Non-Hispanic other	5	(31.3)	0	(0)	0	(0)	2	(11.1)	7	(10.9)
Asian	1	(6.3)	0	(0)	0	(0)	2	(11.1)	3	(4.7)
American Indian or Alaska Native	3	(18.8)	0	(0)	0	(0)	0	(0)	3	(4.7)
Education										
Less than high school	4	(20.0)	2	(12.5)	1	(11.1)	2	(10.5)	9	(14.1)
High school graduate	4	(20.0)	8	(50.0)	4	(44.4)	7	(36.8)	23	(35.9)
Greater than high school	11	(55.0)	6	(37.5)	4	(44.4)	10	(52.7)	31	(48.4)
Unreported	1	(5.0)	0	(0)	0	(0)	0	(0)	1	(1.6)

**Table 2:** PHA resident and staff perceptions about barriers to SFH policy compliance and enforcement.

Domains	Sample Quotes
Domain 1: Outer Setting	
PHA partnerships	“We’re going to be in the next year trying to think of new ways to partner with people who are both into health care and community space we will certainly work to integrate all the different, healthy-home topics around that, so smoke-free alongside with the pest management and the mold work” (Staff #2).
Marijuana policy	“If you’re going to ban smoking, you have to ban the people who are smoking marijuana. It’s permeating the house the same way” (Smoker, FG #6), “Y’all talking about cigarette smoke, [but] if somebody is smoking marijuana and it’s coming in my apartment,’ that’s not a problem”? (Nonsmoker, FG #1) “[Marijuana use] is blatantly in the open, and nobody cares anymore” (Staff #4).
**Domain 2: Intervention characteristics**	“I just don’t think no one could come in my house and tell me what to do, when I pay my rent” (Smoker, FG #2). “Each person has the right to do whatever they please. I can’t say anything to them about it” (Nonsmoker, FG #3). “People have rights. And you sort of invading their privacy when you tell a tenant you can’t smoke in your apartment” (Staff #5).
**Domain 3: Individual characteristics**	“That no-smoking policy is just another rule to set residents up for eviction” (Smoker, FG#2). “I think it’s unfair. You’re going to lose your apartment just because someone complained about cigarette smoke”? (Nonsmoker, FG #3).
**Domain 4: Inner Setting**	
Interpersonal safety concerns	“You’re creating an enemy when you start the reporting” (Nonsmoker, FG #3). “I wouldn’t dare ask somebody to not smoke” (Nonsmoker, FG #8). “[Staff] are told never, ever confront anybody, not for smoking or anything else” (Staff #5).
Structural and environmental barriers	“They are all close-knit buildings. To move 25 feet away from this one you’re only going back in front of this one. Right in front of the next one” (Smoker, FG #9). “I’m not going outside in a snow blizzard to smoke, and in the rain” (Smoker, FG #6).
Community cohesiveness	“Everybody’s been here for ages. Everybody knows each other, and pretty much respects each other” (Smoker, FG#2). “It’s basically every man for themselves. They [other residents] don’t even bother with rules” (Nonsmoker, FG #3).
Lack of smoking cessation services	“I think it would be helpful if they [PHA] started offering an onsite smoking cessation program” (Smoker, FG #9). “What would be nice if we can get a class for people or maybe go around to give out patches” (RA #3).
Relative priority of SFH policy	“They [PHA] should take some of that energy and enforce the policies that already put in place and leave us alone” (Smoker, FG #4). “You’re saying smoking? But there’re a lot of things inside the apartment that are not being dealt with as well” (Nonsmoker, FG #1).
Compatibility	“A lot of property managers feel like they’re overwhelmed, and this is just one more thing that they have to deal with” (Staff #2).
**Domain 5: Implementation Process**	
Planning and community engagement	“This [roll out] could have worked better and been a more positive experience for us had the PHA staff first come in and done this focus group before, and along with some smoking cessation” (Smoker, FG #4). “They could’ve made us more aware by providing more information. And make a list of pros of why you shouldn’t smoke in your apartment” (Smoker, FG #9). “Get out more information and more material to the people” (Nonsmoker, FG #8).
Enforcement	“Housing doesn’t enforce that policy” (Nonsmoker, FG #1). “Their [Housing’s] own employees walking in and out of the building with a cigarette in their mouth. Who are you to tell me not to smoke”? (Smoker, FG #6). “The housing staffs themselves smoke at the front. So why would they implement that rule, if they are the first ones to break it” (Nonsmoker, FG #7). “I’m not going to go into somebody’s house to see whether or not they’re smoking”. (Staff #4)

Notes. FG: focus group. RA: resident association. PHA: public housing authority. SFH: smoke-free housing.

**Table 3:** Nonsmoker reports of satisfaction with and exposure to the SFH policy.

	Pre-Launch (April–July 2018) N = 153		Post-Launch (May–September 2019) N = 130		*p*-Value
	*n*	(%)	*n*	(%)	
Support for NYCHA SFH policy	146	(94.8)	125	(96.2)	0.655
Satisfaction with NYCHA’s introduction of SFH policy	123	(81.5)	91	(70.5)	0.033
Satisfaction with enforcement of SFH policy	89	(60.1)	62	(48.8)	0.047
Which of the following have you experienced in the past 6 months related to SFH policy?					
Saw signs, posters or other materials about the policy in building	78	(50.7)	62	(47.7)	0.593
Received an invitation or seen posters/flyers about meetings to discuss the policy	54	(36.1)	24	(18.5)	0.001
Attended resident meetings where this policy was discussed	14	(9.1)	12	(9.2)	0.999
Submitted a complaint about others smoking in the building	22	(14.3)	28	(21.5)	0.170
Complained directly to a smoker	42	(27.3)	39	(30.0)	0.695
Heard about new smoking cessation services available	22	(14.3)	32	(24.6)	0.071

Notes. NYCHA: New York City Housing Authority. SFH: smoke-free housing.

## Data Availability

All relevant data is contained within the article.

## Supplementary Materials

The following are available online at https://www.mdpi.com/article/10.3390/ijerph182312565/s1, Table S1: Checklist of the Standards for Reporting Qualitative Research (SRQR).
