# Supplementary material for: Implementing the Federal Smoke-Free Public Housing Policy in New York City: Understanding Challenges and Opportunities for Improving Policy Impact
Source: Int J Environ Res Public Health. 2021 Nov 29;18(23):12565. doi: 10.3390/ijerph182312565 (PMC8656672; doi:10.3390/ijerph182312565)
Supplement: Supplementary file 1 [file ijerph-18-12565-s001.zip › ijerph-1450499-SM.pdf]

Table S1: Checklist of the Standards for Reporting Qualitative Research (SRQR)

Section	SRQR	How the manuscript adheres to the reporting standards
Title	S1 Concise description of the nature and topic of the study Identifying the study as qualitative or indicating the approach (e.g., ethnography, grounded theory) or data collection methods (e.g., interview, focus group) is recommended	We described the topic of the study.
Abstract	S2 Summary of key elements of the study using the abstract format of the intended publication; typically includes background, purpose, methods, results, and conclusions.	We summarized key elements of the study in the abstract.
Introduction	S3 Description and significance of the problem/phenomenon studied; review of relevant theory and empirical work; problem statement<br><br>S4 Purpose of the study and specific objectives or questions	We described our research question and a review of relevant literature.<br><br>We described the purpose of the study and specific research question.
Methods: Qualitative approach and research paradigm	S5 Qualitative approach (e.g., ethnography, grounded theory, case study, phenomenology, narrative research) and guiding theory if appropriate; identifying the research paradigm (e.g., postpositivist, constructivist/interpretivist) is also recommended; rationale	We included a 'Conceptual framework' section in the Methods.
Methods: Researcher characteristics and reflexivity	S6 Researchers' characteristics that may influence the research, including personal attributes, qualifications/experience, relationship with participants, assumptions, and/or presuppositions; potential or actual interaction between researchers' characteristics and the research questions, approach, methods, results, and/or transferability	We described who conducted the focus groups and key informant interviews. We did NOT describe interviewer characteristics (e.g., bias, assumptions, reasons and interests in the research topic) as we believe they are not relevant.
Methods: Context	S7 Setting/site and salient contextual factors; rationale	We described the study setting.
Methods: Sampling strategy	S8 How and why research participants, documents, or events were selected; criteria for deciding when no further sampling was necessary (e.g., sampling saturation); rationale	We described the eligibility criteria, recruitment method, and sample size.
Methods: Ethical issues pertaining to human subjects	S9 Documentation of approval by an appropriate ethics review board and participant consent, or explanation for lack thereof; other confidentiality and data security issue	We stated in the Methods section that "The study was approved by New York University Grossman School of Medicine's Institutional Review Board."
Methods: Data collection methods	S10 Types of data collected; details of data collection procedures including start and stop dates of data collection and analysis, iterative process, triangulation of sources/methods, and modification	We described the qualitative data collection procedures.

	of procedures in response to evolving study findings; rationale.	
Methods<br>Setting and description of sample	S12 Number and relevant characteristics of participants, documents, or events included in the study; level of participation (could be reported in results)	Table 1 includes the number and characteristics of participants. We did NOT include participants' gender and age in Table 1.<br><br>We described the year and months that the qualitative interviews took place.
Methods<br>Data collection and data processing	S10 Types of data collected; details of data collection procedures including (as appropriate) start and stop dates of data collection and analysis, iterative process, triangulation of sources/methods, and modification of procedures in response to evolving study findings; rationale<br><br>S11 Description of instruments (e.g., interview guides, questionnaires) and devices (e.g., audio recorders) used for data collection; if/how the instrument(s) changed over the course of the study<br><br>S13 Methods for processing data prior to and during analysis, including Transcription.	We described the data collection methods (semi-structured interviews and focus groups).<br><br>We described interview guides including topics and questions.<br><br>We described the qualitative format: semi-structured interview and focus groups.<br><br>We described that data was audio recorded and the durations of the interviews/focus groups. We described that data was transcribed verbatim.<br><br>The transcripts were NOT returned to the participants. The interview guide was NOT piloted, nor did we use field notes. Neither was necessary in our study.<br><br>We did NOT repeat interviews to ensure data saturation.
Methods<br>Research ethics and data management	S9 Ethical issues pertaining to human subjects.<br><br>S13 Data entry, data management and security, verification of data integrity, data coding, and anonymization/deidentification of excerpts	We described that oral informed consent was obtained from all participants prior to the interview.
Methods<br>Data analysis	S14 Process by which inferences, themes, etc., were identified and developed, including the researchers involved in data analysis; usually references a specific paradigm or approach; rationale<br><br>S15 Techniques to enhance trustworthiness and credibility of data analysis (e.g., member checking, audit trail, triangulation); rationale	We described the number of data coders, the process by which we identified themes in the data. We described that we used a deductive analysis strategy.<br><br>We described the software used for data coding and analysis.

Results	S16 Main findings (e.g., interpretations, inferences, and themes); might include development of a theory or model, or integration with prior research or theory<br><br>S17 Evidence (e.g., quotes, field notes, text excerpts, photographs) to substantiate analytic findings	Quotations were presented to substantiate the analytical findings. We believed that the results are clearly presented.<br><br>It is NOT possible to identify each participant in the focus groups, which is not considered relevant.
Discussion Interpretation of findings	S18 Short summary of main findings; explanation of how findings and conclusions connect to, support, elaborate on, or challenge conclusions of earlier scholarship; discussion of scope of application/generalizability; identification of unique contribution(s) to scholarship in a discipline or field	We discussed the main findings within the context of the literature and identify our contributions. We offered implications for future research and practice.
Discussion Limitations	S19 Trustworthiness and limitations of findings	We described study strengths and limitations.
Other	S20 Potential sources of influence or perceived influence on study conduct<br>and conclusions; how these were managed<br><br>S21 Sources of funding and other support; role of funders in data collection, interpretation, and reporting	We declared no conflicts of interests. We declared that the study was supported by National Cancer Institute (NCI) at the National Institutes of Health (R01CA220591). NCI played no role in study design, data collection and analysis, manuscript writing, or the decision to submit the paper for publication.
